# Dental Medicine

**Published:** 1889-07

**Authors:** 


### Bibliographical.

---

DENTAL MEDICINE.—The two former editions of "Dental Medicine" have been noticed with approval in the *Dental Cosmos* as they appeared. Their exhaustion in so short a time is evidence of the favor with which the book was received. The present volume contains nearly one hundred pages of new matter. An improvement have been made in the arrangement of a portion of the subject-matter, and considerable additions have been made to a number of the topics. Quite a number of medicinal agents with dental uses have been added, and also new formulæ. We can repeat with emphasis the commendation of former editions, and the claim that as an educator and as a work of reference it should be in the hands of every student and every practitioner of dentistry.—*Dental Cosmos*.

